# Enticing Dimples: A Novel Observational Study Comparing the Preexisting Landmarks for Facial Dimple Creation Using 3D-Scanned Facial Images

**DOI:** 10.1055/s-0044-1801772

**Published:** 2025-01-13

**Authors:** Lena Elizabath David, Madhubari Vathulya, Smruti Srinivasan, Yogesh Bahurupi, N Nasida Fathima

**Affiliations:** 1 Department of General Surgery, All India Institute of Medical Sciences, Rishikesh, Uttarakhand, India; 2 Department of Burns and Plastic Surgery, All India Institute of Medical Sciences, Rishikesh, Uttarakhand, India; 3 Department of Community and Family Medicine, All India Institute of Medical Sciences, Rishikesh, Uttarakhand, India

**Keywords:** dimpleplasty, 3D image, landmarks for dimple creation, facial ratios

## Abstract

**Background** Dimpleplasty, a cosmetic procedure for creating dimples, is gaining popularity. However, the ideal site for dimple creation remains subjective. This study aimed to determine the optimal dimple placement using a three-dimensional (3D) model, reviewing panelists' preferences based on facial proportions and average ratings.

**Materials and Methods** A 3D model was used to assess three dimple placement points: Point A (intersection of the external canthus and Cupid's bow), point B (Khoo Boo–Chai point), and point C (site based on smile dynamics). Panelists reviewed these points on faces with different proportions using three facial ratios: ratio 1 (Tr-Me / lc r-lc l), ratio 2 (Tr-Nos / Nos-Me), and ratio 3 (Me-Lip / Lip-Nos). Panelists provided average ratings for each site based on aesthetic appeal relative to these ratios.

**Results** Point A received higher ratings for faces with a lower ratio 1 (wider faces). Point B was preferred in faces with higher ratio 1 (elongated faces) and lower ratio 2, receiving the highest average rating for balanced aesthetics. Point C was favored in faces with higher ratios 2 and 3, indicating dominance in the mid and lower facial thirds. Point B had the highest average rating overall.

**Conclusion** These results emphasize the importance of considering facial proportions for optimal dimple placement and individualized cosmetic outcomes. Simulation software like the one that we have used in our study, may further help in patient counseling before determining the site of dimpleplasty.

## Introduction

Dimples over the face are a dynamic facial feature often linked with youth and beauty. Anatomically, this is believed to be due to a bifid zygomaticus major muscle, which divide to have insertions to the dermis and provide a tethering effect. 1 2 Dimpleplasty is a minimally invasive surgery that involves creating a depression in the dermis, simulating a natural dimple. The increasing demand for dimpleplasty necessitates an understanding of the most aesthetically pleasing and natural-looking site for dimple creation.

Despite the rising interest in dimpleplasty, there is a lack of consensus on where dimples should ideally be placed. Factors such as cheek anatomy, facial symmetry, and cultural preferences contribute to this complexity. Previous studies have predominantly focused on subjective assessments, with very few employing objective metrics such as facial ratios or three-dimensional (3D) models. 4

This study aims to bridge this gap by examining the ideal dimple site using a combination of 3D facial models and panelist evaluations based on facial ratios. The primary goal of this study was to establish a standardized approach to dimpleplasty, considering the aesthetic influence of facial proportions. A group of panelists evaluated different dimple positions on 3D models, which allowed us to investigate patterns in dimple placement preference. Additionally, we examined key facial ratios to determine their relationship with optimal dimple sites.

## Materials and Methods

The study was performed in a single tertiary care hospital after taking requisite approval from the Department Review Board and the Institutional Ethics Committee. Female participants who did not have a dimple on their cheek and willing to participate were included. Subjects with visible scars on the cheek, including dimples, and those who denied consent for participation were excluded from the study. Based on these inclusion criteria, 60 participants were selected with a diverse range of facial structures and ethnic backgrounds to ensure that the findings were applicable across a variety of populations. High-resolution 3D facial scans were generated for each participant using a 3D surface scanner.

**Fig. 1:**
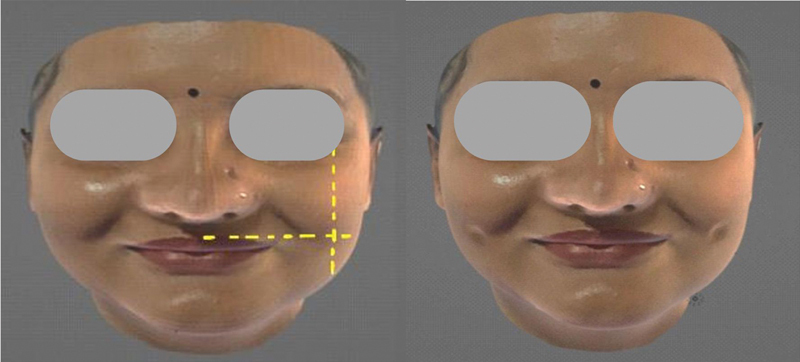
The point of intersection of the perpendicular line dropped forth further from the external canthus and a horizontal line drawn from the highest point of the Cupid bow laterally (point A).

**Fig. 2:**
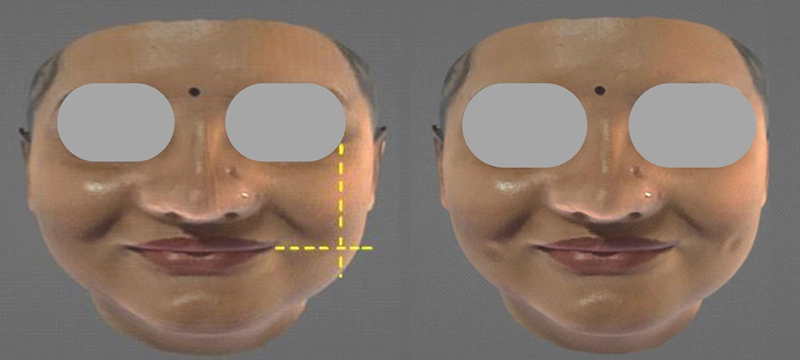
Khoo Boo–Chai (KBC) point: the intersection between a horizontal line drawn from the corner of the mouth and a vertical line dropped from the outer canthi of the eye (point B).

**Fig. 3:**
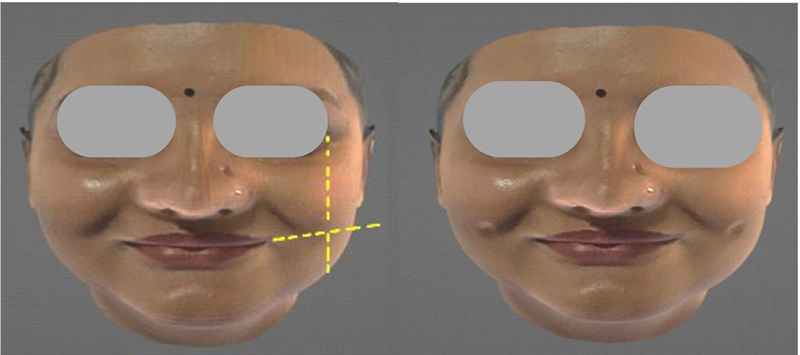
The site at the level of the angle of the mouth or above it according to the vector of the smile and a vertical line at the level of the external canthus (point C).

**Fig. 4:**
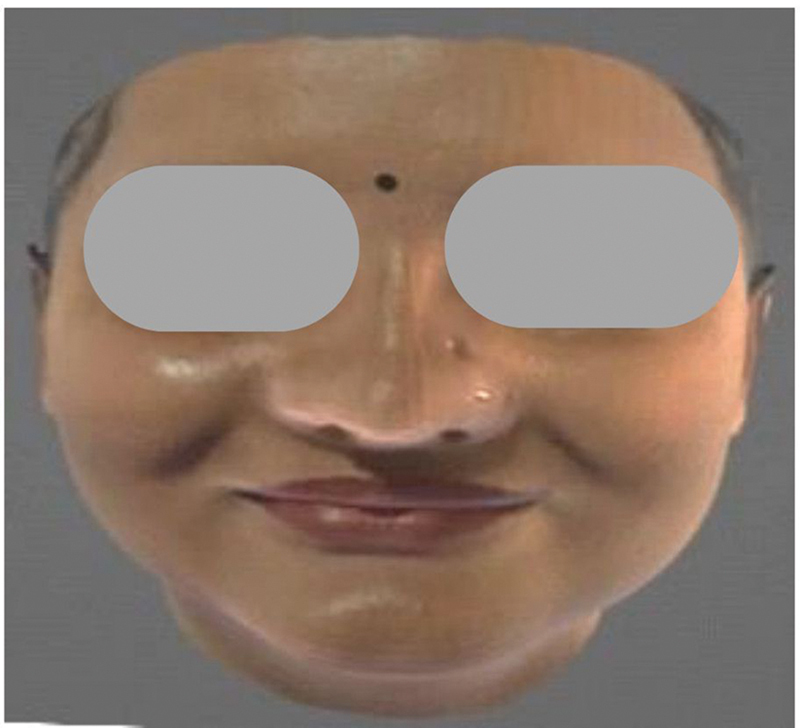
No dimple (unmodified): Images without any dimples.

The participants' faces were scanned using a structured light-based 3D facial scanner to obtain precise measurements of their facial structures. These models were then imported and modified using the special Blender software sculpting tool for further analysis. Four potential sites for dimple placement were created based on anatomical landmarks:

- Point A: The point of intersection of a perpendicular line dropped from the external canthus (outer corner of the eye) and a horizontal line drawn from the highest point of the Cupid's bow laterally 2 ( Fig. 1 ).
- Khoo Boo–Chai (KBC) point (point B): The intersection of a vertical line dropped from the outer canthus of the eye and a horizontal line drawn from the corner of the mouth 3 ( Fig. 2 ).
- Point C: A site at the level of the angle of the mouth or slightly above it, according to the vector of the smile, with a vertical line aligned with the external canthus 5 ( Fig. 3 ).
- No dimple (unmodified): Images without any dimples to serve as control models for comparison ( Fig. 4 ).

A group of four panelists, including plastic surgeons, dermatologists, nonmedical person, and subject were recruited for the study. Each panelist was tasked with assessing the ideal location for dimples on each 3D facial model. The panelists were unaware of the participant's identity to eliminate bias related to gender, ethnicity, or personal attributes. Panelists were asked to assess the aesthetic appeal of dimples placed at each of these sites for every 3D model. They rated the aesthetic quality of each site using a 5-point Likert scale, where 1 was “very unattractive” and 5 was “very attractive.”

**Fig. 5:**
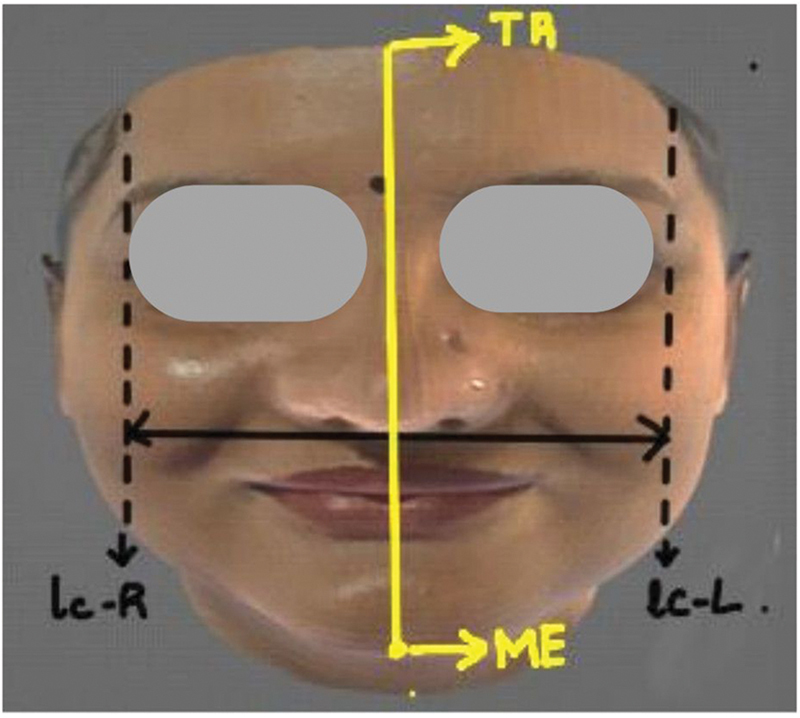
Ratio 1 (Tr-Me / lc-R- lc-L): The ratio between the height of the face (Tr-Me, measured from the trichion to the menton) and the width of the face (lc-R- lc-L).

**Fig. 6:**
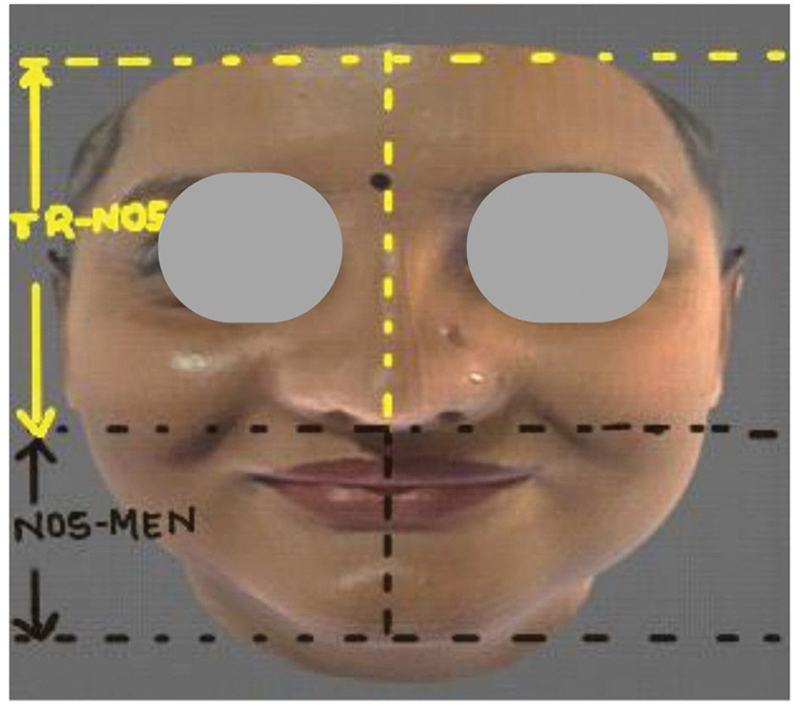
Ratio 2 (Tr-Nos / Nos-Me): The ratio between the distance from the forehead's highest point (trichion) to the nostril (Tr-Nos) and the distance from the nostril to the lowest point on the chin (Nos-Me).

**Fig. 7:**
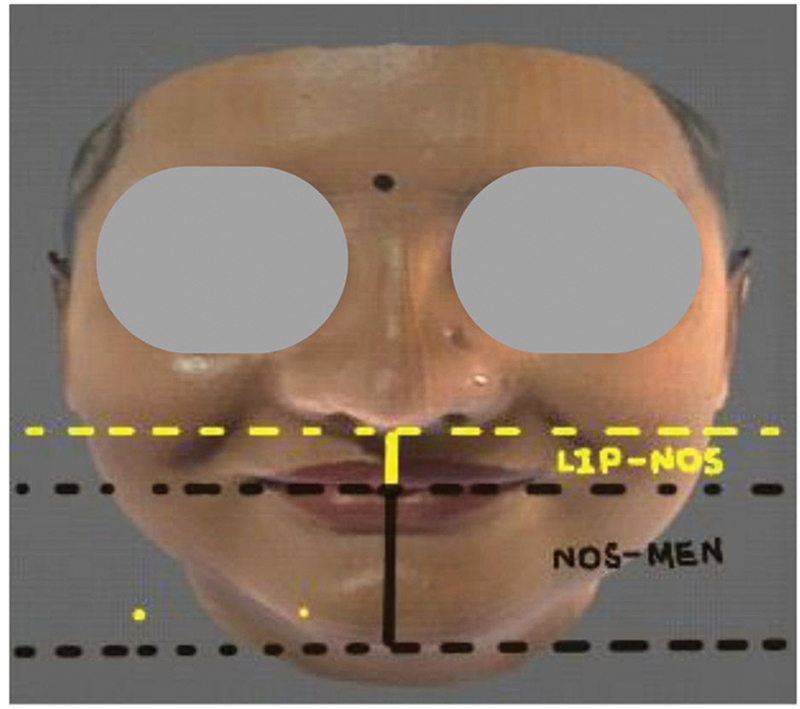
Ratio 3 (Me-Lip / Lip-Nos): The ratio between the distance from the lowest point on the chin (menton) to the point where the upper and lower lips merge (Me-Lip), and the distance from the lip merger point to the outer edge of the nostril (Lip-Nos).

In the photographs, several parameters were also measured like:

- Ratio 1 (Tr-Me / lc-R- lc-L): The ratio between the height of the face (Tr-Me, measured from the trichion to the menton) and the width of the face (lc-R- lc-L) ( Fig. 5 ).
- Ratio 2 (Tr-Nos / Nos-Me): The ratio between the distance from the forehead's highest point (trichion) to the nostril (Tr-Nos) and the distance from the nostril to the lowest point on the chin (Nos-Me) ( Fig. 6 ).
- Ratio 3 (Me-Lip / Lip-Nos): The ratio between the distance from the lowest point on the chin (menton) to the point where the upper and lower lips merge (Me-Lip), and the distance from the lip merger point to the outer edge of the nostril (Lip-Nos) ( Fig. 7 ).

These facial proportions were analyzed to determine their influence on the aesthetic appeal of dimple placement across different sites (points A, B, and C).

The ratings from the panelists were averaged for each site and each model. Descriptive statistics, including means and standard deviations, were calculated for the ratings of each potential dimple site. Additionally, the correlation between facial ratios and dimple placement preferences was analyzed using Pearson's correlation coefficient.

## Results

**Fig. 8:**
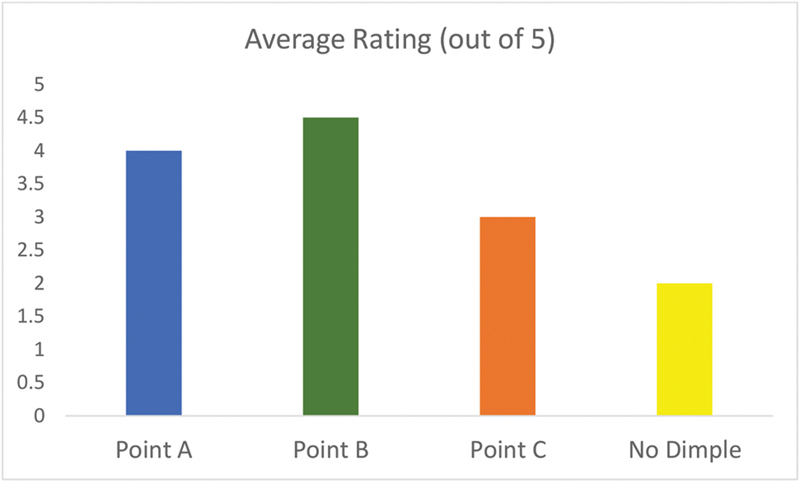
Average rating for dimple sites.

The evaluation by the panelists yielded significant insights into the preferences for the different dimple sites. Point B (KBC) emerged as the most preferred site for dimple creation, with an average rating of 4.5 out of 5. Point A received a slightly lower average score of 4.0 while Point C, located at the level of the mouth's angle, yielded a more mixed response. The panelists rated it an average of 3. For images without any dimple (the control), the average rating for facial aesthetics was 2, serving as a baseline for comparison ( Fig. 8 ).

### Correlation with Facial Proportions: The Correlation between the Dimple Sites and the Facial Ratios Provided Crucial Insights

**Fig. 9:**
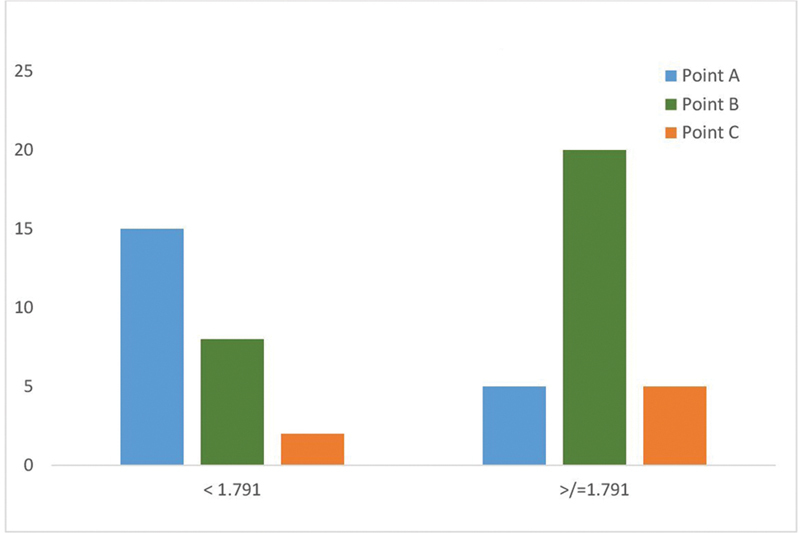
Correlation between ratio 1 and dimple landmarks.

Ratio 1 (Tr-Me / lc-R- lc-L) ( Fig. 9 ): A higher facial height-to-width ratio was positively correlated ( *r* = 0.68) to point B. Conversely, point A was preferred for individuals with a wider facial width relative to height ( *r* = –0.54). The cutoff being ≥ 1.791. That is, for individuals with ratio 1 ≥ 1.791 (higher ratio 1), panelists preferred point B for dimple placement, while for those with ratio 1 < 1.791 (lower ratio 1), point A was preferred.

**Fig. 10:**
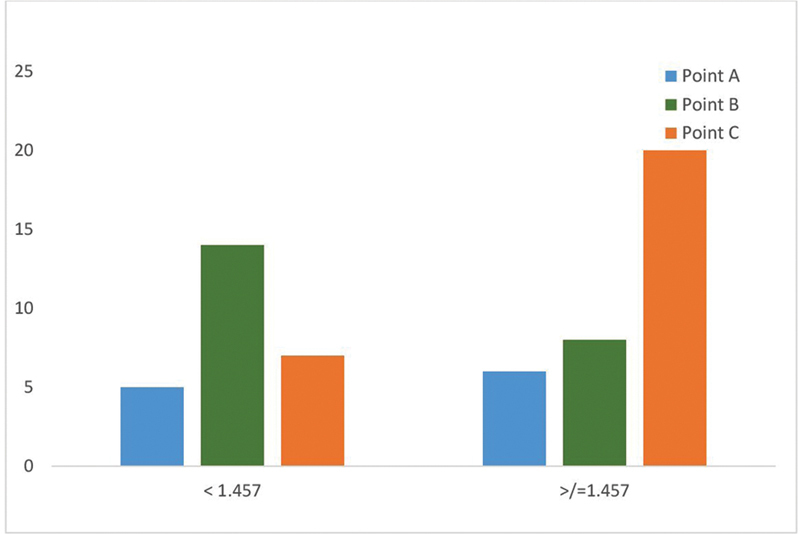
Correlation between ratio 2 and dimple landmarks.

Ratio 2 (Tr-Nos / Nos-Me) ( Fig. 10 ): This ratio reflects the relationship between the upper and the lower face, Higher ratio 2 (≥ 1.457), indicating longer vertical dimensions, favored point C ( *r* = 0.61). Lower ratio 2 (< 1.457), representing shorter vertical facial proportions, preferred point B (KBC point) ( *r* = –0.45).

**Fig. 11:**
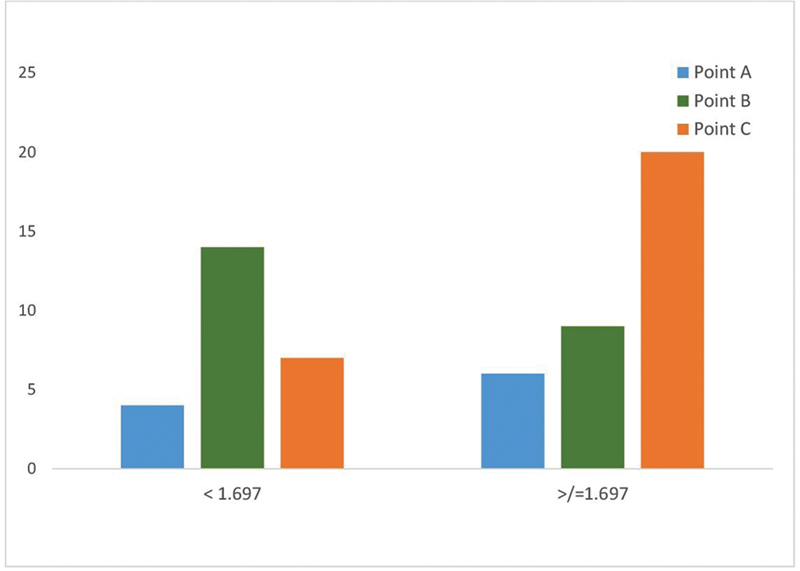
Correlation between ratio 3 and dimple landmarks.

Ratio 3 (Me-Lip / Lip-Nos) ( Fig. 11 ): For individuals with ratio 3 ≥ 1.697 (higher ratio 3), point C was preferred by the panelists ( *r* = 0.48), while for those with ratio 3 < 1.697 (lower ratio 3), point B (KBC point) was favored for individuals with shorter chin-to-lip distances ( *r* = –0.48).

## Discussion

Dimpleplasty has gained importance and has become a common cosmetic procedure due to the enhancement of facial attractiveness and smile. Although the frequency of this procedure is increased, not much literature is available regarding the ideal landmark for the location of dimple. The commonly used reference point, the KBC point, can be traced back to 50 years. Koo Boo Chai in 1962, conducted a study of 500 females and concluded that most of the dimples are located at a point of intersection between a perpendicular line dropped from the lateral canthi of the eye and a horizontal line drawn out from the angle of the mouth. 3 Apart from the KBC point, surgeons have identified many other reference points for creating dimple over years.

In 2012, Lari and Panse suggested two methods for deciding the site of dimple creation: (1) using the point of intersection of a perpendicular line dropped from the lateral canthus and a horizontal line drawn from the highest point of the Cupid's bow and (2) marking the site of maximum depression when the patients suck in their cheeks as the location for the dimple. 2 El-Sabbagh recommended making a dimple at a point that is level with or above the angle of the mouth, according to the vector of smile. 5 Shaker et al suggested a dimple by asking the patient to smile and then marking the dimple site 2 to 2.5 cm lateral to the nasolabial fold at the same level or slightly above the angle of the mouth. 6 Jones et al used the transoral buccinator-pexy technique, to create a malar dimple using the landmark of 1.5 to 2 cm above the point of intersection of the line joining the lateral commissure and base of the ear lobule with a perpendicular line from the lateral canthus. 7 Though many reference points have been described for the creation of dimples, we were unable to find reports in the available literature that correlated with varying facial ratios, when choosing the optimal location for dimples. The varying measurements of the face influence the location of the dimple and can be used objectively for planning before surgery. 9 10

This study's findings demonstrate that the KBC point (point B) is the most favored dimple site overall, achieving the highest aesthetic ratings. Its alignment with both facial landmarks (lateral canthus and mouth corner) and facial proportions suggests that it offers the most natural and balanced outcome for a broad range of individuals. This site appears to universally enhance facial aesthetics without overwhelming or distorting facial harmony. Point A, although less preferred overall, gained favor for individuals with wider faces, supporting the negative correlation between ratio 1 (face height to width) and the appeal of point A. For individuals with a broader facial structure, placing the dimple closer to the center of the face (point A) helped achieve balance and proportional harmony. Point C, positioned near the angle of the mouth, was favored for individuals with higher ratio 2 values, indicating that this site was more suitable for people with elongated faces.

This study demonstrates that facial ratios play a crucial role in determining the optimal site for dimple placement. Each ratio had specific cutoff points, indicating preferences for different dimple locations

Ratio 1 (Tr-Me / width), representing the height-to-width ratio of the face, was a strong determinant of dimple placement preferences. For individuals with higher ratio 1 (≥ 1.791), indicating a longer and narrower face, point B (KBC point) was favored by most panelists. This preference is based on the ability of point B to balance vertical elongation. Placing dimples at point B draws attention toward the lateral areas of the face, mitigating the perception of narrowness while adding a subtle, natural contour to the smile line. 11

Conversely, for those with lower ratio 1 (< 1.791), representing shorter and wider faces, point A was preferred. Point A, closer to the center of the face near the Cupid's bow, reduces the perception of width by focusing attention on the midline. This finding aligns with the aesthetic principle that centralizing facial features can enhance the overall harmony of wider facial structures.

Ratio 2 (Tr-Nos / Nos-Me), which reflects the balance between the upper and lower thirds of the face, also influenced dimple placement preferences. In individuals with a higher ratio 2, the upper third of the face (forehead to nasal base) dominates the lower third (nose to chin). Placing dimples at Point C helps compensate for the shorter lower face by extending the visual width of the lower third. Point C is located laterally near the angle of the mouth, which enhances the horizontal dimension of the lower third, helping to offset the visual dominance of the upper third. By shifting attention away from the vertical length of the upper third, point C creates a balanced overall appearance. 11

Individuals with lower ratio 2 (< 1.457), indicating a more compact vertical dimension, showed a strong preference for point B. This placement, aligning with the outer canthus and the corner of the mouth, enhances facial symmetry and harmonizes with the shorter vertical height, as seen in earlier studies. 12

Ratio 3 (Me-Lip / Lip-Nos), reflects the balance of the lower face. Patients with a higher ratio 3 tend to have a longer lower third of the face, specifically between the chin and the lips. By placing the dimple laterally at point C, a horizontal line of interest is created that visually breaks up the vertical length of the chin-to-lip area. This helps distribute facial features more evenly across both the vertical and horizontal axes, leading to a more proportional look. When the smile is activated, the dimple at point C aligns naturally with the broader, expansive movement of the mouth. 13 This contributes to a more harmonious and attractive smile, making the face appear well-proportioned.

A lower ratio 3 (< 1.697) indicates that the distance between the chin and lips is shorter relative to the lip-to-nose distance, creating a more compact lower facial third. A shorter distance between the chin and lips (lower ratio 3) can give the appearance of a more compact or compressed lower face. Dimples placed at point B, farther from the central axis, help “expand” the lower facial third laterally and lower, which visually softens the compactness and creates a more harmonious look. This placement spreads the attention across a wider area of the face, preventing the focus from being solely on the small vertical distance between the chin and lips.

Thus,

Point B is preferred in individuals whose lower half of the face is shorter.
Point A is preferred in population with wider face.
Point C is preferred for faces with longer lower proportions.

There were a few limitations in our study. First is the small sample size. Second, there is no debate that the different degrees of smile enhance the aesthetic outlook of the face. The degree of smile has not been quantified and hence standardization is not possible.

## Conclusion

The results of this study provide valuable insights for plastic surgeons performing dimpleplasty. The optimal dimple location should be determined not only by visual preferences but also by considering individual facial proportions. By considering these facial ratios, plastic surgeons can achieve more personalized, aesthetically pleasing outcomes in dimpleplasty procedures. This evidence-based approach allows for a tailored method of dimple placement, ensuring harmony between the created dimple and the patient's unique facial structure. Future studies could investigate the influence of cultural preferences on dimple placement and further refine these guidelines for use across diverse populations.
